# Challenge model of TNF_α_ turnover at varying LPS and drug provocations

**DOI:** 10.1007/s10928-019-09622-x

**Published:** 2019-02-18

**Authors:** Felix Held, Edmund Hoppe, Marija Cvijovic, Mats Jirstrand, Johan Gabrielsson

**Affiliations:** 1 Fraunhofer-Chalmers Centre, Chalmers Science Park, Gothenburg, Sweden; 2 Department of Mathematical Sciences, Chalmers University of Technology and University of Gothenburg, Gothenburg, Sweden; 3 Grünenthal GmbH, Aachen, Germany; 4 Department of Biomedical Sciences and Veterinary Public Health, Swedish University of Agricultural Sciences, Box 7028, 75007 Uppsala, Sweden

**Keywords:** Target biology, Kinetic-dynamic modelling, Challenge tests, Experimental design, Non-linear mixed effects modelling

## Abstract

A mechanism-based biomarker model of TNF_α_-response, including different external provocations of LPS challenge and test compound intervention, was developed. The model contained system properties (such as *k*_*t*_, k_*out*_), challenge characteristics (such as *k*_*s*_, k_*LPS*_, K_*m, LPS*_, S_*max*_, SC_*50*_) and test-compound-related parameters (*I*_*max*_, IC_*50*_). The exposure to test compound was modelled by means of first-order input and Michaelis–Menten type of nonlinear elimination. Test compound potency was estimated to 20 nM with a 70% partial reduction in TNF_α_-response at the highest dose of 30 mg·kg^−1^. Future selection of drug candidates may focus the estimation on potency and efficacy by applying the selected structure consisting of TNF_α_ system and LPS challenge characteristics. A related aim was to demonstrate how an exploratory (graphical) analysis may guide us to a tentative model structure, which enables us to better understand target biology. The analysis demonstrated how to tackle a biomarker with a baseline below the limit of detection. Repeated LPS-challenges may also reveal how the rate and extent of replenishment of TNF_α_ pools occur. Lack of LPS exposure-time courses was solved by including a biophase model, with the underlying assumption that TNF_α_-response time courses, as such, contain kinetic information. A transduction type of model with non-linear stimulation of TNF_α_ release was finally selected. Typical features of a challenge experiment were shown by means of model simulations. Experimental shortcomings of present and published designs are identified and discussed. The final model coupled to suggested guidance rules may serve as a general basis for the collection and analysis of pharmacological challenge data of future studies.

**Electronic supplementary material:**

The online version of this article (10.1007/s10928-019-09622-x) contains supplementary material, which is available to authorized users.

## Introduction

Tumour necrosis factor alpha (TNF_α_) is a pro-inflammatory cytokine associated with the pathogenesis of several immune-mediated diseases, such as rheumatoid arthritis and Crohn disease [1]. Since TNF_α_ release is a typical response to a variety of inflammatory mediators, it became an important biomarker for various diseases mediated by inflammation [2]. Free TNF_α_ is almost undetectable in blood of healthy organisms. However, pro-inflammatory challengers can induce TNF_α_ expression and release of soluble TNF_α_ after proteolytic cleavage of a precursor molecule by TNF_α_-converting enzyme TACE/ADAM-17 [7]. Experimentally, the effect of the inflammatory mediators is studied in vitro in whole blood assays or in vivo after intravenous administration of lipopolysaccharides LPS, where the challenger causes a rapid but transient release of TNF_α_ [3, 6]. The in vivo LPS-challenge models are commonly utilized in drug discovery to identify and characterize anti-inflammatory drugs [4, 5]. However, experimental design will have a great impact on the results, particularly for drug-related pharmacodynamic parameters such as potency and efficacy [6]. In a typical in vivo LPS challenge experiment, only TNF_α_ and test-compound concentrations are measured over time after a single LPS dose. The fact that the exposure to LPS concentrations is difficult to quantify causes a modelling problem. The question arises of how to define the stimulatory input of TNF_α_-response. Therefore, some of the current models use an LPS-stimulated biophase input [6].

Several models of LPS-induced TNF_α_-response have been proposed, including, to name just the most recent: (1) linearly stimulated turnover in combination with a series of transit compartments [6]; (2) a lag-time approach to pre-cursor-determined TNF_α_ production [10, 12, 24]; (3) soluble TNF_α_ with a time-dependent turnover rate [11, 12, 25]; (4) a quadratic function for TNF_α_ production [26]; (5) an inhibitory *I*_*max*_ model of TNF_α_ [27]; (6) a nonlinear FAA-driven stimulatory model with lag-time [28]. All of these models lack to a varying extent a quantitative description of delayed onset, saturable intensity and extended duration of LPS-induced TNF_α_-response following several dose levels of both LPS and test compound.

**Fig. 1:**
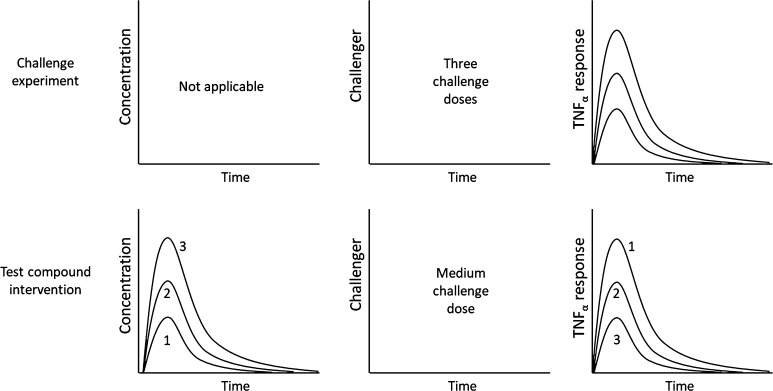
Schematic presentation of the two studies incorporated into the analysis. Upper row: three LPS challenge doses (3, 30 and 300 µg·kg^−1^ of LPS) were given in Study 1 and the TNF_α_-response was measured. No time courses are available for LPS. Bottom row: the middle challenge dose (30 µg·kg^−1^ of LPS) was selected for three groups of rats that received 0.3, 3 and 30 mg·kg^−1^ of test-compound A in Study 2

Three different LPS challenges (Study 1) and three inhibitory test-compound doses (Study 2) are investigated from a macro-pharmacological perspective using TNF_α_-response as a biomarker of target behavior (Fig. 1). Test-compound A is a selective inhibitor of phosphodiesterase (PDE) type 4 isoforms. The PDE4 isoforms have been shown to be involved in the LPS-induced TNF_α_ release using genetic knockouts, and with the marketed pan-PDE4 inhibitors apremilast and roflumilast [30, 31].

The goal was therefore to identify the determinants of target biology related to TNF_α_ turnover by means of pooling data from two preclinical studies in rats. This was done in order to answer the question: Will multiple LPS and test-compound provocations help in simultaneously characterizing TNF_α_ system behavior, LPS challenge characteristics and test-compound properties, as suggested earlier. The analysis was tailored to derive a kinetic-dynamic model of TNF_α_-response, which has potential in discovery data analyses. Therefore, a meta-analysis was performed on available data from two separate studies on TNF_α_-response after multiple LPS and test compound interventions. For this purpose, a mixed-effects approach was a useful tool. Typically, if an accurate and precise estimate of the pharmacodynamic properties of a test compound is sought, time-series analyses of challenger- and biomarker-time data are necessary. Erosion of data, resulting in the single-point assessment of drug action after a challenge test, should be avoided. This is particularly relevant for situations where one expects time-curve shifts, functional adaptation, impact of disease, or hormetic concentration-response relationships to occur [6].

## Materials and methods

### Chemicals

Lipopolysaccharides (LPS) from Escherichia coli 0111:B4 was obtained from Sigma (Product number L4391; the same batch 036M4070V was used for both studies). The test-compound A was synthesized at Grunenthal, Aachen, Germany, and the purity of the batch used in this study was ≥ 95%. The physico-chemical properties of test compound A are presented in Table 1. Test-compound A was developed as an inhibitor of PDE4. The rat TNF_α_ Quantikine ELISA kit was purchased from R&D systems (SRTA00, Batches P143557, P118837, and 339837). All other reagents and chemicals were of analytical grade and were obtained from standard vendors.

**Table 1:** Physico-chemical properties of compound A

Parameter	Value
Molecular weight	< 500 g·mol^−1^
cLogP	< 2.5
PSA	< 80 Å^2^
Solubility	> 10 µmol·L^−1^ at pH 7.4

### Animals

The studies were conducted in male Sprague–Dawley rats, approximately 210–260 g of body weight, purchased from Vital River Laboratory Animals Co. LTD. All rats were housed in groups under 12 h light/dark cycle with ad libitum access to food and water. During the study, animals were not fasted, but no food was provided prior to dosing until 3 h after drug dosing. All animals were handled in strict accordance with the Guide for the Care and Use of Laboratory Animals in an AAALAC-accredited facility. All animal studies were approved by an established Institutional Animal Care and Use Committee (IACUC).

### Design of in vivo studies

LPS was dissolved in saline at 0.0006, 0.006, and 0.06 mg·mL^−1^ and 5 mL·kg^−1^ of the solutions were dosed intravenously via foot dorsal vein injection to give doses of 3, 30 and 300 µg·kg^−1^, respectively. Test-compound A was suspended in 1% HPMC (5 mPa s, Colorcon) and 0.5% Tween 80 (Sigma) in water at concentrations of 0.06, 0.6, and 6 mg·mL^−1^. Test-compound A was administered at a volume of 5 mL·kg^−1^ by oral gavage, resulting in doses of 0.3, 3 and 30 mg·kg^−1^, respectively.

**Fig. 2:**
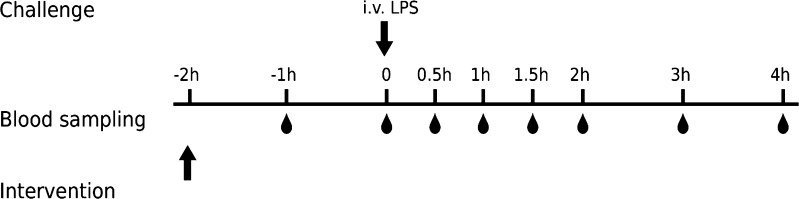
Schematic presentation of the designs of Study 1 and 2. Arrows denote time of test-compound and LPS administration. Blood droplets denote harvesting of plasma samples for assessment of test-compound concentrations and TNF_α_-response, respectively. Test compound was only administered in Study 2 and no blood sample at − 1 h was taken in Study 1

Forty-eight normal male Sprague–Dawley rats were used in the LPS-induced TNF_α_-response model in the absence (Study 1) or presence (Study 2) of test-compound A (Fig. 1). The animals were randomly divided into eight groups (n = 6). In Study 1, four groups of animals were given increasing intravenous doses of LPS (0, 3, 30 and 300 μg·kg^−1^ LPS). In Study 2, four groups of animals received a fixed intravenous dose of LPS challenger of 30 μg·kg^−1^ and increasing oral doses of test compound (0, 0.3, 3 and 30 mg·kg^−1^ compound A). Test compound was administered two hours before the challenge with LPS. Blood samples were drawn for quantification of Test-compound A and TNF_α_ before dosing of test compound (at − 2 h) and at − 1, 0, 0.5, 1, 1.5, 2, 3, and 4 h after LPS dosing (Fig. 2). Blood samples were collected into EDTA-2K tubes via tail vein or cardiac puncture for terminal bleeding. Samples were stored on ice and centrifuged at 2000×*g* for 5 min at 4 °C within 15 min after sampling. Each plasma sample was divided into two aliquots, one for LC-MS/MS analysis to measure test compound concentrations, and one for ELISA analysis to measure the biomarker TNF_α_ concentrations. Until quantification, the plasma samples were stored at −70 °C after snap-freezing of plasma in dry ice.

Table 2 summarizes the experimental design of the two studies. Study 1 was conducted to characterize the dose-response-time relationships of the TNF_α_-release after LPS challenge and to define an appropriate LPS challenge dose. Study 2 investigated the inhibition of this response by Test-compound A using a fixed LPS challenge dose and three inhibitory test-compound doses. Full response time courses for TNF_α_ were obtained and analyzed by modelling. The test-compound concentrations over time were measured as well, but the actual exposure to LPS could not be quantified due to the nature of LPS, which consists of a poorly defined mixture of different components of the bacterial cell wall.

**Table 2:** Overview of experimental designs of the two individual studies

Study	Challenge compound	Animal model	Test-compound	PD effect biomarker	Designs
1	LPS	Rat	–	TNF_α_	Three LPS challenge doses (3, 30 and 300 µg·kg^−1^); lacks challenger time course(s); no drug intervention
2	LPS	Rat	A	TNF_α_	One LPS challenge dose (30 µg·kg^−1^); lacks challenger time course(s); three test-compound intervention doses (0.3, 3 and 30 mg·kg^−1^)

### Bioanalytical methods

#### Quantification of TNF_α_ concentrations by ELISA

TNF_α_ concentrations in plasma were quantified with the rat TNF_α_ Quantikine ELISA Kit (R&D Systems, SRTA00) according to the instructions provided in the kit, using seven calibrations standards ranging from 12.5 to 800 ng·L^−1^. The measured concentrations of the quality controls were all in the range as specified in the kit instruction and showed CV % < 20%. The lower limit of quantification (LLOQ) was 12.5 ng·L^−1^ and lower values were reported as “<LLOQ” and excluded from subsequent evaluation and parameter estimation.

#### Quantification of test-compound A concentrations by LC-MS/MS

For the quantification of the test compound, acetonitrile which contained dexamethasone as internal standard was added to plasma prepared from the blood samples for protein precipitation. Supernatants were injected onto a C18 reversed phase column for LC-MS/MS analysis. The UPLC separation was carried out using a gradient elution in H_2_O containing 0.025% formic acid/1 mM NH_4_OAc (mobile phase A) and methanol that contained 0.025% formic acid/1 mM NH_4_OAc (mobile phase B). The analytes were quantified on an API5500 mass spectrometer using multiple reaction monitoring with appropriate mass transitions. Each set of samples was run together with two calibration sets containing nine non-zero standard concentrations covering a range of range from 1 to 3000 nM. Quality controls of 3, 500, and 2400 nM were interspersed between the samples. The calculated concentrations of the calibration samples and quality controls were within ± 15% of the nominal values (20% at LLOQ) for at least 75% and 67% of the samples, respectively. Concentrations below 80% of the LLOQ (i.e. below 0.8 nM) were reported as “<LLOQ” and excluded from subsequent evaluation and parameter estimation.

### Pharmacokinetic and pharmacodynamic models

#### Test compound kinetics

**Fig. 3:**
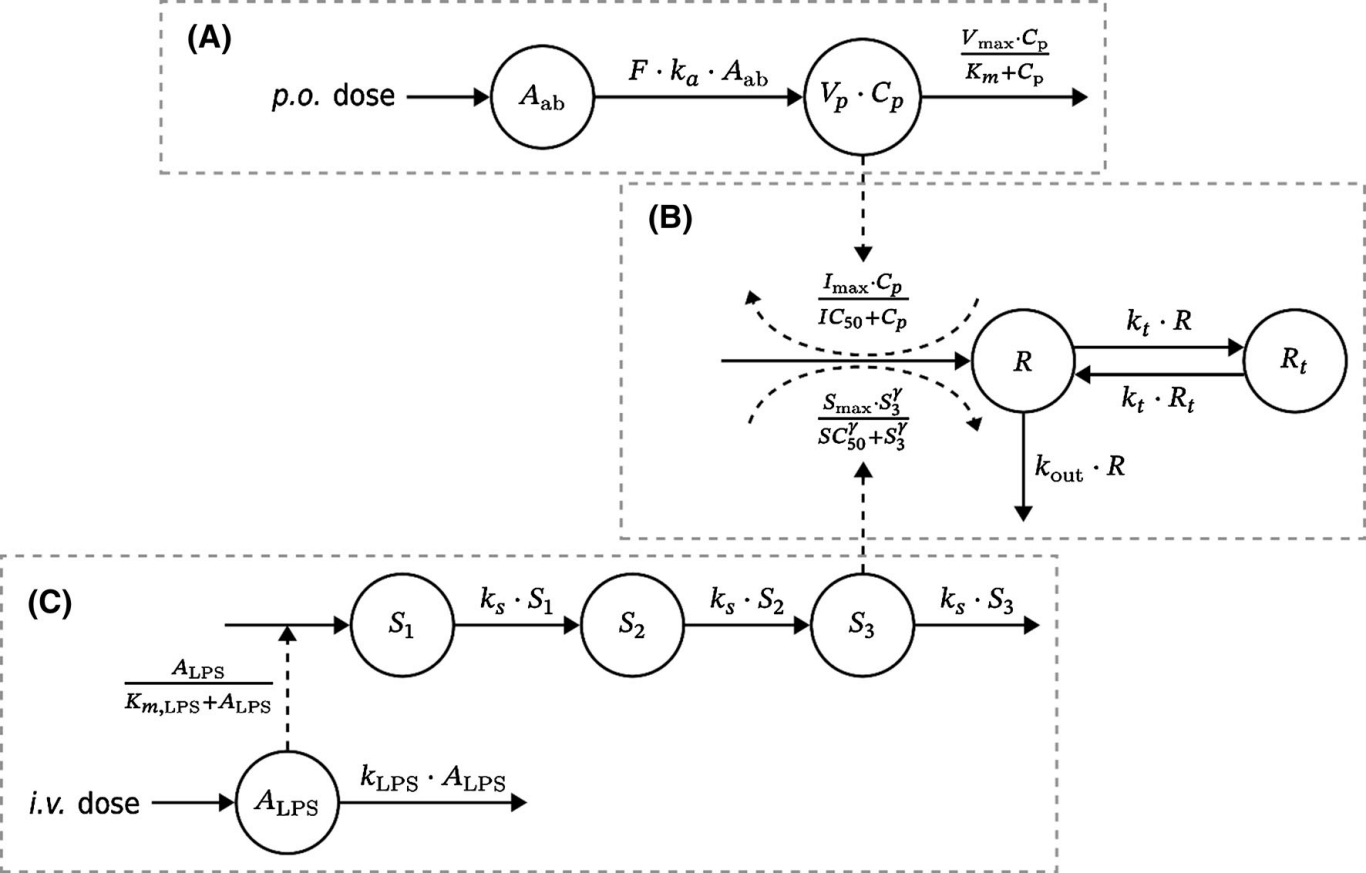
Schematic presentation of the kinetic and dynamic model. Solid lines symbolize mass transfer and dashed lines stand for control streams. Upper row A: Kinetic model of test compound disposition after oral administration. Here, *A*_*ab*_ and *C*_*p*_ denote, respectively, amount and concentration in the gut and central plasma compartment. The volume of the latter is denoted by *V*_*p*_. *F* and *k*_*a*_ are the bioavailability and the absorption rate of the test compound. *V*_*max*_ and *K*_*m*_ are the maximum elimination and Michaelis–Menten constant. Middle row B: Turnover model for the TNF_α_-response. TNF_α_ is divided into compartments *R* and *R*_*t*_. Here, *k*_*t*_ and *k*_*out*_ denote the first-order transfer rate between compartments and elimination rate from the system. TNF_α_ turnover is stimulated by LPS challenge from part C and inhibited by test compound kinetics from part A. Here, *I*_*max*_ is maximum inhibitory capacity of the test-compound and *IC*_*50*_ its potency, *S*_*max*_ is maximum stimulatory capacity, *γ* is a Hill exponent and *SC*_*50*_ is the potency of LPS challenge. Bottom row C: Model of LPS challenge. A first-order biophase *A*_*LPS*_ describes LPS after intravenous administration with first-order elimination rate *k*_*LPS*_. LPS non-linearly stimulates a signal chain (*S*_*1*_ to *S*_*3*_) with Michaelis–Menten constant *K*_*m*_, and signal transfer—as well as elimination rate *k*_*s*_. A more detailed description of the principal parts of the model and their behavior are discussed in appendix

The impact of test compound on the TNF_α_-response is shown conceptually in Fig. 3a and b. The first-order loss of test compound from the gut is given by Eq. 1.

1 $$ \frac{{{\text{d}}A_{ab} }}{{{\text{d}}t}}\, = \, - \,k_{a} A_{ab} $$

The plasma exposure to test compound was then described by a one-compartment model with first-order oral input and Michaelis–Menten elimination.

2 $$ V_{p} \cdot \frac{{{\text{d}}C_{p} }}{{{\text{d}}t}}\, = \,{\text{F}} \cdot k_{a} \cdot A_{ab} \, - \,\frac{{V_{max} \cdot C_{p} }}{{K_{m} \, + \,C_{p} }} $$

*A*_*ab*_ denotes amount of test compound in the gut*, C*_*p*_ exposure to drug in plasma, *k*_*a*_ the first-order absorption rate constant, *V*_*max*_ maximum rate of elimination, *K*_*m*_ the Michaelis–Menten constant, and *V*_*p*_ volume of distribution. The bioavailability *F* was set to unity.

#### LPS challenge model

The impact of the LPS challenge on the TNF_α_-response is shown conceptually in Fig. 3b and c. The intravenous LPS dose is injected into plasma as a bolus and cleared from plasma via first-order elimination.

3 $$ \frac{{dA_{LPS} }}{dt}\, = \, - \,k_{LPS} A_{LPS} $$

The level of LPS in plasma triggers a series of transduction compartments with a saturable process *A*_*LPS* _*/ (K*_*m, LPS *_+* A*_*LPS*_*)*. The *S*_*3*_ signal acts on the build-up of TNF_α_-response via stimulatory action (*S(S)*_*3*_). The transduction of LPS-induced signal from *S*_*1*_ through *S*_*3*_ is given by Eq. 4.

4 $$ \begin{aligned} \frac{{{\text{d}}s_{\textit{1}} }}{{{\text{d}}t}}\, & = \,k_{s} \cdot \left( {\frac{{A_{LPS} }}{{K_{m,\,LPS} \, + \,A_{LPS} }} - S_{\textit{1}} } \right) \\ \frac{{{\text{d}}s_{\textit{2}} }}{{{\text{d}}t}}\, & = \,k_{s} \cdot \left( {S_{\textit{1}} \, - \,S_{\textit{2}} } \right) \\ \frac{{{\text{d}}s_{\textit{3}} }}{{{\text{d}}t}}\, & = \,k_{s} \cdot \left( {S_{\textit{2}} \, - \,S_{\textit{3}} } \right) \\ \end{aligned} $$

*A*_*LPS*_ is LPS amount in the biophase and *S*_1_ to *S*_3_ are a chain of transduction compartments which act as signaling compartments. LPS is thought to be eliminated with rate constant *k*_*LPS*_. Signal *S*_1_ is stimulated non-linearly by LPS with Michaelis–Menten constant *K*_*m*_. Rate constant *k*_*s*_ describes transfer of signal across *S*_1_ to *S*_3_ and loss from system.

#### TNF_α_ turnover model

Figure 3b shows conceptually the TNF_α_ turnover *R* and the impact of both the LPS challenge and the test compound kinetics on the TNF_α_-response. The dynamics of TNF_α_-response is divided into a central *R* and a peripheral *R*_*t*_ pool governed by a first-order inter-compartmental rate constant *k*_*t*_, in order to capture the post-peak bi-phasic decline of response. The irreversible loss of TNF_α_ occurs from its central compartment via a first-order rate process *k*_*out*_*· R*.

The stimulatory action via *S*_*3*_ of LPS-induced challenge is given by Eq. 5.

5 $$ {S}\left( {{S}_{\textit{3}} } \right) = \,\frac{{S_{ max } \cdot S_{\textit{3}}^{\gamma } }}{{SC_{\textit{50}}^{\gamma } + S_{\textit{3}}^{\gamma } }} $$

*S*_*max*_ is the maximum LPS stimulatory production rate of TNF_α_, and *SC*_*50*_ is the corresponding transducer concentration *S*_*3*_ where 50% of maximum rate occurs. The inhibitory action of test compound *I(C*_*p*_*)* on build-up of response is.

6 $$I(C_p) = 1 - \frac{I_{max} \cdot C_p}{IC_{\textit{50}} + C_p}$$

The structure of Eq. 6 allows a partial *I*_*max*_ inhibitory effect of the test compound. The *IC*_*50*_ parameter denotes the concentration of test compound resulting in 50% of maximal test-compound inhibitory capacity.

Equations 5 and 6 are then combined in Eq. 7 describing the TNF_α_-response in the central *R* and peripheral *R*_*t*_ compartments.

7 $$ \begin{aligned} \frac{{{\text{d}}R}}{{{\text{d}}t}} & & = S(S_{\textit{3}} ) \cdot I(C_{p} ) - k_{out} R + k_{t} \cdot \left( {R_{t} - R} \right) \\ \frac{{{\text{d}}R_{t} }}{{{\text{d}}t}} & = k_{t} \cdot (R - R_{t} ) \\ \end{aligned} $$

*S*_*max*_ is the maximum stimulatory capacity, *SC*_*50*_ concentration of *S*_*3*_ at 50% of maximum stimulation, *γ* a Hill exponent, *I*_*max*_ maximum inhibitory capacity by test compound and *IC*_*50*_ test compound potency. Neither *S*_*1*_, *S*_*2*_ or *S*_*3*_, nor TNF_α_-response display any baseline concentrations in the proposed model. Without any stimulation from LPS there is no TNF_α_-response to inhibit with test compound.

The determinants of the TNF_α_-response at equilibrium are given by Eq. 8.

8 $$ R_{\text{eq}} = \frac{1}{{k_{out} }} \cdot S(S_{\textit{3}} ) \cdot I(C_{p} ) = \frac{1}{{k_{out} }} \cdot \frac{{S_{ max } \cdot S_{\textit{3}}^{\gamma } }}{{SC_{\textit{50}}^{\gamma } + S_{\textit{3}}^{\gamma } }} \cdot \left( {1 - \frac{{I_{ max } \cdot C_{p} }}{{IC_{\textit{50}} + C_{p} }}} \right) $$

This expression is presented as a 3D-plot in Appendix 2 using the final parameter estimates from regressing TNF_α_ response time data.

### Data analysis

Non-linear mixed-effects modelling (NLME) [13] was used to regress the model in Fig. 3 to TNF_α_-response data and to capture inter-individual variability (IIV). The number of animals was small (18 and 17 subjects in Study 1 and 2, respectively). Therefore, the IIV estimation was restricted to *V*_*max*_, *k*_*LPS*_, *SC*_*50*_, *k*_*out*_, *I*_*max*_ and *IC*_*50*_ (See Appendix). Residual variance of compound exposure was modelled with an additive error model on the log-scale and for response concentrations with a proportional error model.

Model parameters were estimated using Monolix [20], including stochastic approximation for the determination of standard errors. In step 1, parameters in Eqs. 3–5 and 7 were based on TNF_α_-responses from Study 1. The pharmacokinetic parameters in Eqs. 1 and 2 were estimated from test compound data from Study 2. The pharmacokinetic parameters were then fixed together with systems parameters from Step 1, and *I*_*max*_ and *IC*_*50*_ were estimated from Study 2 data. Further computational details can be found in Appendix 1.

## Results

### Experimental data

**Fig. 4:**
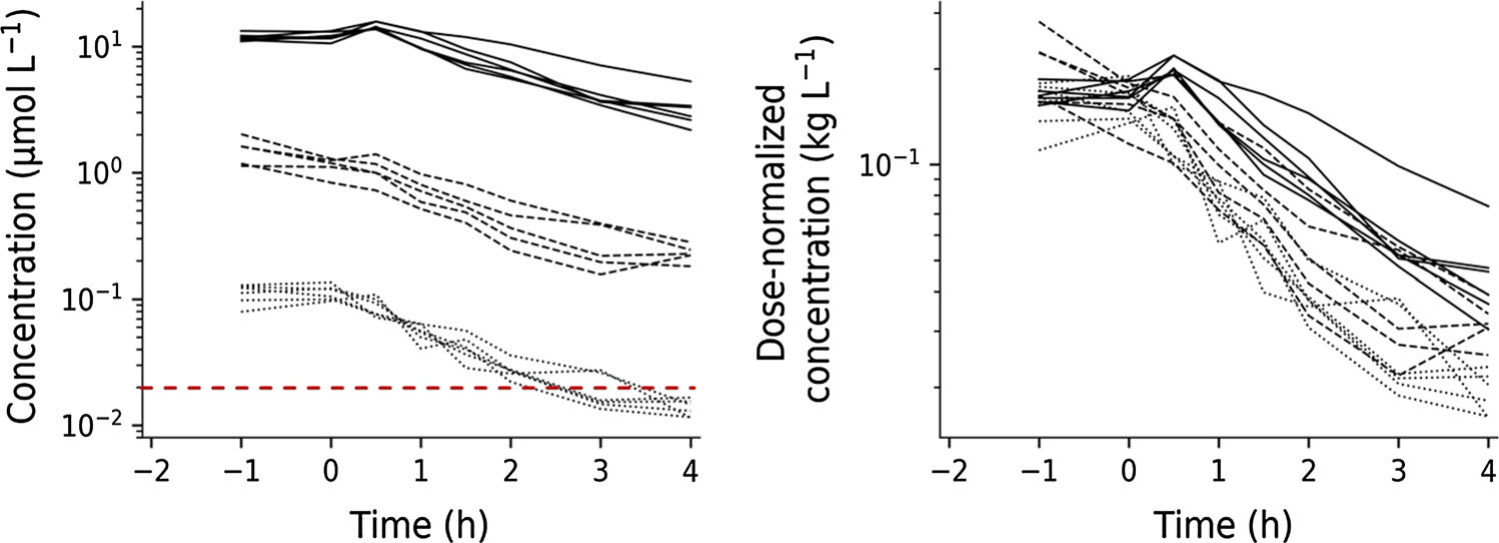
Left: semi-logarithmic plot of concentration–time data of test-compound A at three oral doses (0.3, 3 and 30 mg·kg^−1^, shown as dotted, dashed and solid lines, respectively) in Sprague–Dawley rats from Study 2. Test compound was administered 2 h before intravenous challenge with LPS. The dashed red horizontal line represents the model-predicted test compound potency of about 20 nM. Right: Dose-normalized test compound concentrations plotted versus time

Figure 4 shows the plasma concentration–time course of test compound (left) and dose-normalized plasma concentrations (right). The exposure to test compound increases disproportionately with increasing doses of test compound, which suggests nonlinear elimination with increasing oral doses. There is also a weak tendency of a longer terminal half-life with increasing oral doses. This nonlinearity was captured by Eq. 1.

**Fig. 5:**
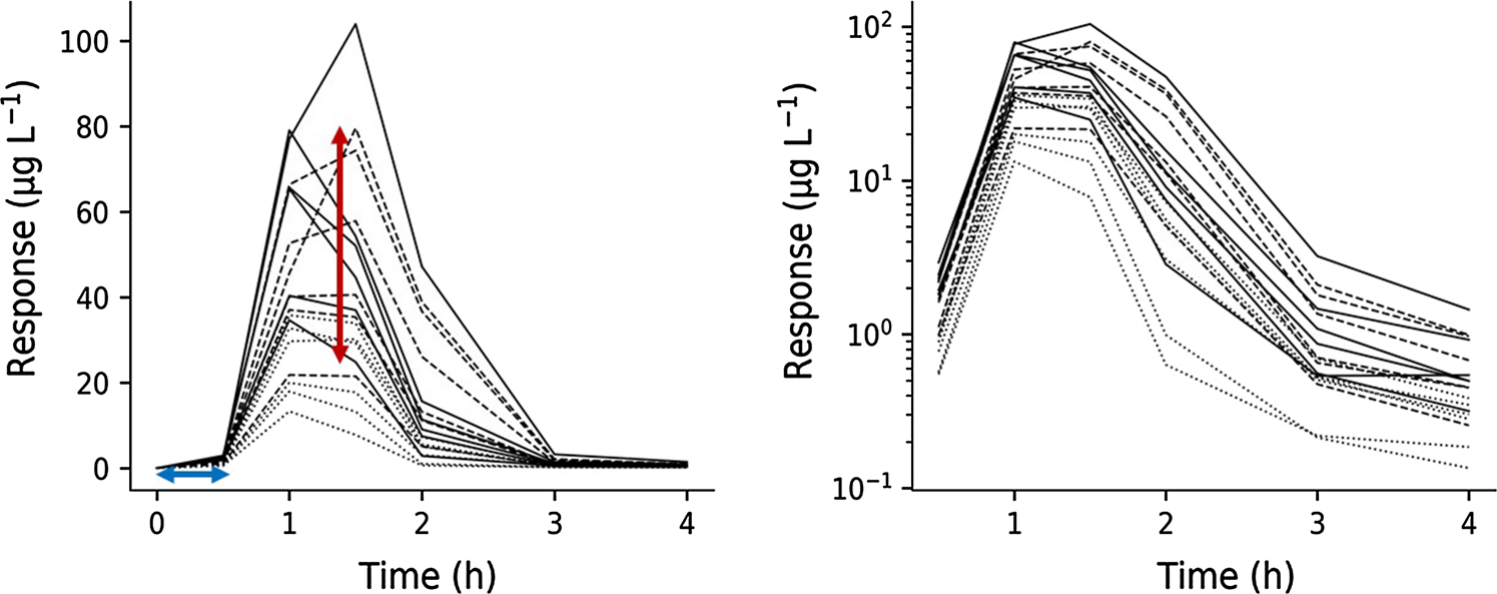
Left: TNF_α_-response time courses at increasing intravenous LPS challenge (3, 30 and 300 μg·kg^−1^ LPS, shown as dotted, dashed and solid lines, respectively) in Sprague–Dawley rats from Study 1. The blue horizontal double arrow represents the initial time delay in onset of response, and the red vertical double arrow, the 20–80 range in TNF_α_ peak-response of the 30 μg·kg^−1^ LPS challenge. Right: Semi-logarithmic plot of the same TNF_α_-response time courses (Color figure online)

The TNF_α_-response following three different intravenous LPS challenge doses of 3, 30 and 300 µg·kg^−1^ is shown in Fig. 5. TNF_α_ data display a 30 min time-delay in onset of response independently of challenge dose (Fig. 5 left). Additionally, TNF_α_-response time courses show a bi-phasic post-peak decline (Fig. 5 right). This motivated the two-compartment structure for the TNF_α_-response.

**Fig. 6:**
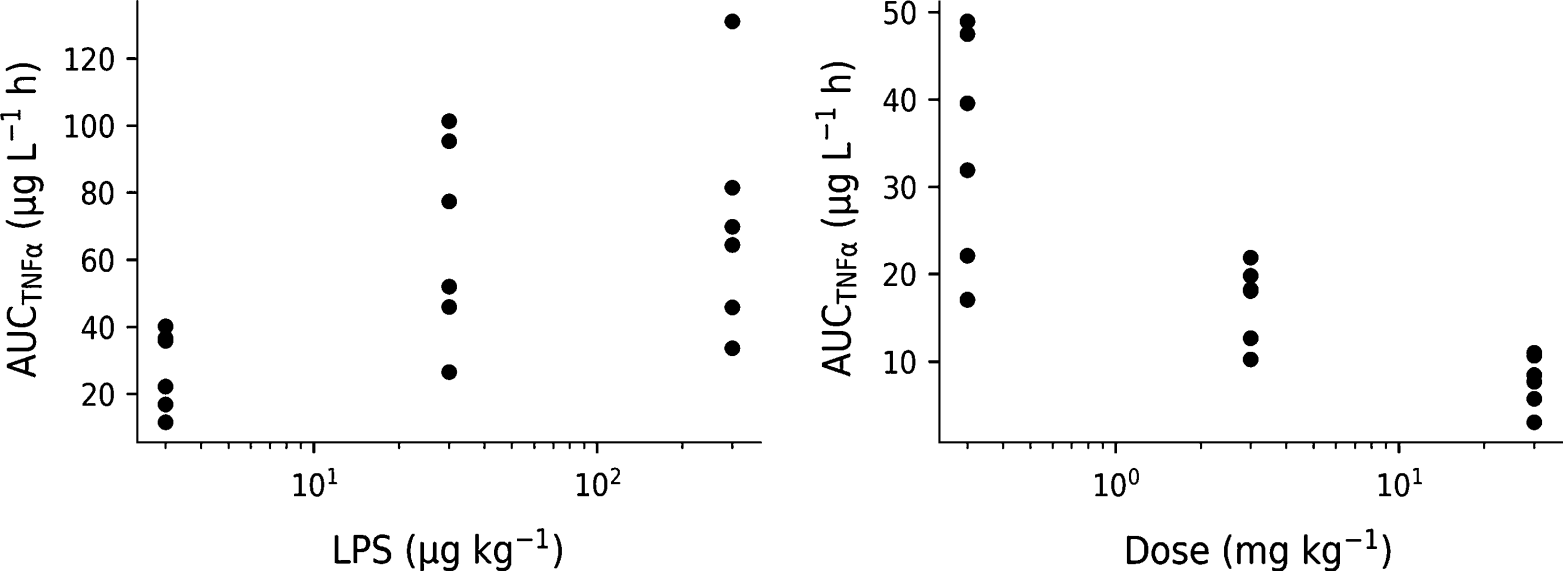
Left: Area under the TNF_α_-response plotted versus increasing LPS challenge doses (3, 30 and 300 μg·kg^−1^ LPS) from Study 1. Right: Area under the TNF_α_-response plotted versus increasing oral doses of test compound (0.3, 3 and 30 mg·kg^−1^ test-compound A) and a fixed intravenous LPS challenge with 30 μg kg^−1^ from Study 2

The areas under the TNF_α_-response time curves are plotted *versus* LPS challenge dose in Fig. 6 (left, Study 1), as are the areas under the TNF_α_-response time at a fixed LPS challenge dose of 30 µg·kg^−1^ but increasing test compound doses of 0.3, 3 and 30 mg·kg^−1^ (right, Study 2). The exploratory analysis shows that both increasing LPS doses and increasing test compound doses have an opposite nonlinear impact on the TNF_α_ response.

**Fig. 7:**
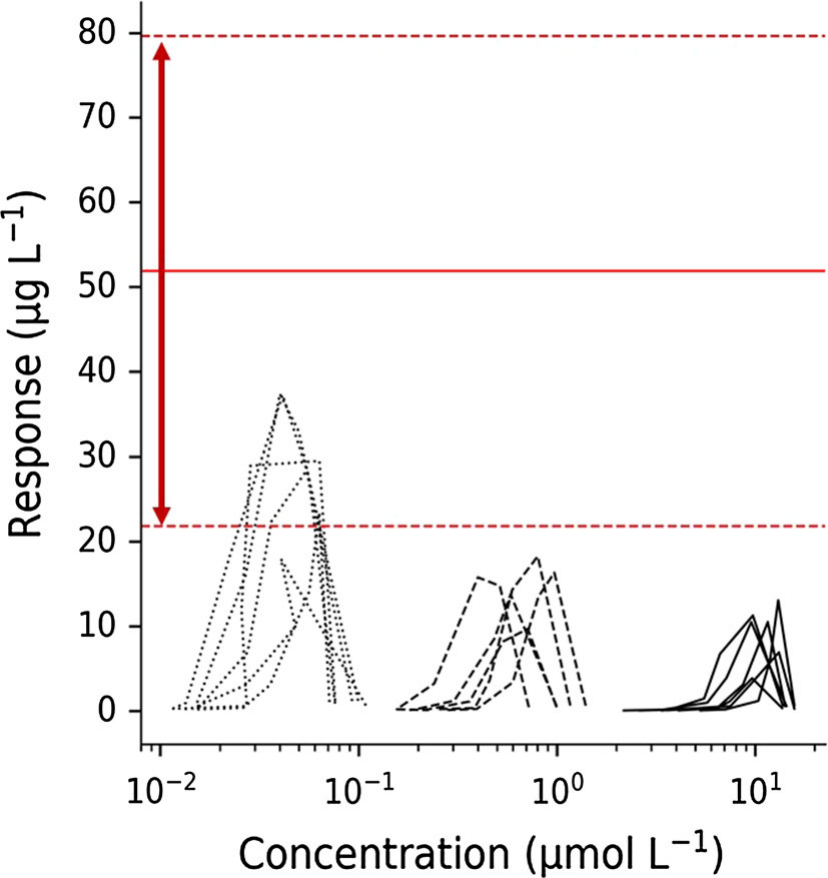
Hysteresis plot of individual TNF_α_-response plotted versus A concentrations following a fixed intra-venous LPS challenge dose (30 μg·kg^−1^) and increasing oral doses of test-compound A (0.3, 3 and 30 mg·kg^−1^). The upper and lower dashed horizontal lines represent the TNF_α_ peak response range in vehicle control animals given only a 30 μg·kg^−1^ LPS challenge dose (Study 1)

Figure 7 shows TNF_α_ response *versus* test compound concentrations for the fixed 30 µg·kg^−1^ LPS challenge and three test compound interventions (0.3, 3 and 30 mg kg^−1^ Compound A, Study 2) superimposed on the peak TNF_α_ response range (horizontal red dashed lines) of 30 µg·kg^−1^ LPS challenge (Study 1). There is a 50% reduction in TNF_α_ peak response already at the lowest test compound dose, suggesting that efficacious test compound concentrations fall within the 10–100 nM range.

The TNF_α_ model is mathematically described by Eqs. 1–7. The first-order input and Michaelis–Menten-output were obtained from separately regressing concentration-time data of test compound. A biophase compartment was included to mimic the time courses of LPS in plasma. The 30 min LPS dose-independent time delay of TNF_α_-response was captured by simultaneously combining a series of transit compartments with a nonlinear stimulatory term of transit compartment *S*_*1*_. The latter varied between zero and unity and allowed the same time of onset of action for the TNF_α_-response for all LPS doses. The intensity of TNF_α_-response showed saturation with increasing LPS doses. This was modelled by means of a nonlinear stimulatory function with its own LPS-potency parameter *SC*_*50*_, driven by the last transit compartment *S*_*3*_. The bi-phasic post-peak decline of TNF_α_-response was captured by means of a two-compartment (central *R* and peripheral *R*_*t*_) model. TNF_α_-response time data of Study 1 were regressed after increasing LPS challenge doses (3, 30 and 300 µg·kg^−1^ LPS). Regression of TNF_α_-response time data of Study 2 after increasing oral test compound doses (0.3, 3 and 30 mg·kg^−1^ Compound A) with a fixed intravenous LPS challenge (30 µg·kg^−1^) was then done as a last step to get potency *IC*_*50*_ and maximum inhibitory capacity *I*_*max*_ of test compound.

### Model regression

#### TNF_α_ during LPS challenge: study 1

**Fig. 8:**
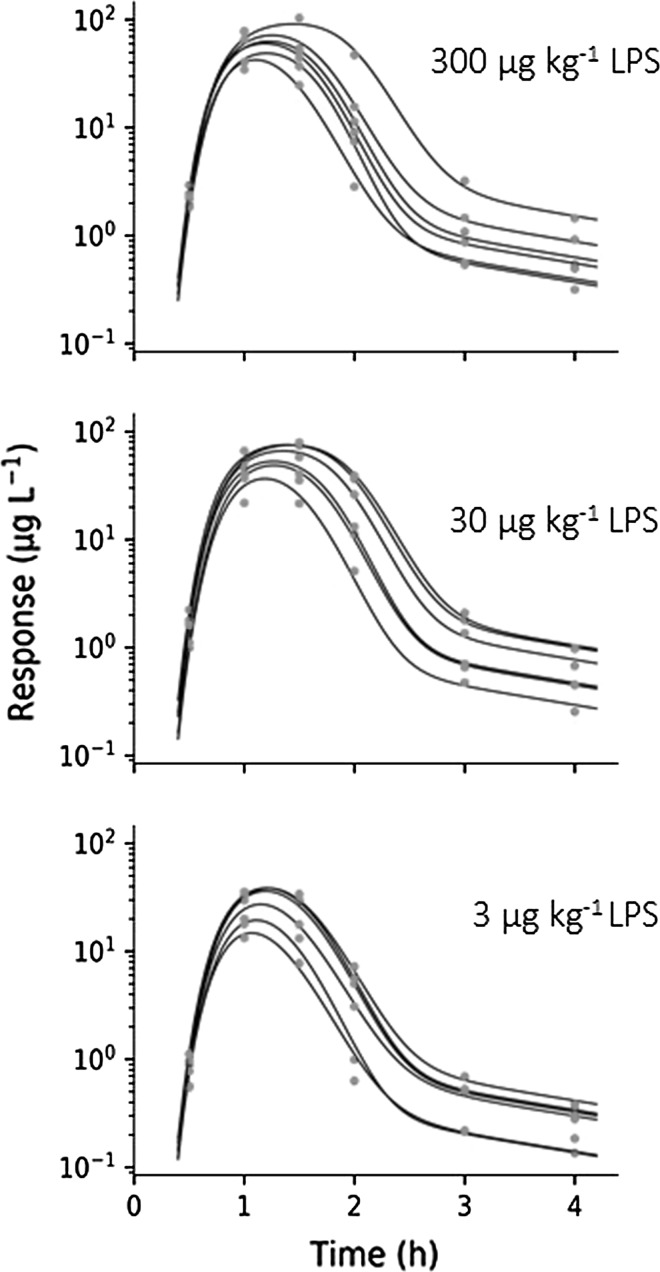
Observed concentrations (red dots) and predicted response time courses (solid lines) of TNF_α_-response for all subjects in Study 1. LPS challenge was 3 µg·kg^−1^ (upper), 30 µg·kg^−1^ (middle) and 300 µg·kg^−1^ (lower) (Color figure online)

Equations 3 and 4 captured the TNF_α_-response at all LPS challenges (Fig. 8) and revealed system properties (such as *k*_*t*_*, k*_*out*_) and challenge characteristics (such as *k*_*s*_*, k*_*LPS*_*, K*_*m, LPS*_*, S*_*max*_*, SC*_*50*_). Future selection of potential drug candidates may focus the estimation on potency and efficacy applying the selected framework while keeping system fixed.

Experimental data show a 30 min time lag in onset coupled to a slight peak-shift in TNF_α_-response at increasing LPS doses, which suggests a nonlinear stimulation of TNF_α_ release. The final parameter estimates and their precision (CV%) are shown in Table 3. The predicted half-life of TNF_α_-response was less than 10 min. The elimination rate constant of LPS from the biophase compartment, the transit compartment rate constant and the fractional turnover rate of TNF_α_-response were all short and fell in the same range (with half-lives of 5, 13 and 7 min, respectively).

**Table 3:** Final pharmacodynamic model estimates, their CV% and IIV and IIV CV% as well as resulting half-life

Parameter	Units	Final estimate	CV%	IIV%	IIV CV%	Half-life (min)
*k*_*LPS*_	h^−1^	8.36	29	30.4	19	5
*k*_*s*_	h^−1^	3.28	8.1	–	–	13
*K*_*m,LPS*_	µg·kg^−1^	0.0789	19	–	–	
*S*_*max*_	ng·L^−1^·h^−1^	6·10^5^	12	–	–	
*SC*_*50*_	–	0.469	14	9.0	42	
*γ*	–	3.79	2.5	–	–	
*k*_*out*_	h^−1^	5.65	30	14.8	34	7
*k*_*t*_	h^−1^	0.419	37	–	–	100

#### TNF_α_ during a fixed LPS challenge coupled to varying test compound intervention: study 2

**Fig. 9:**
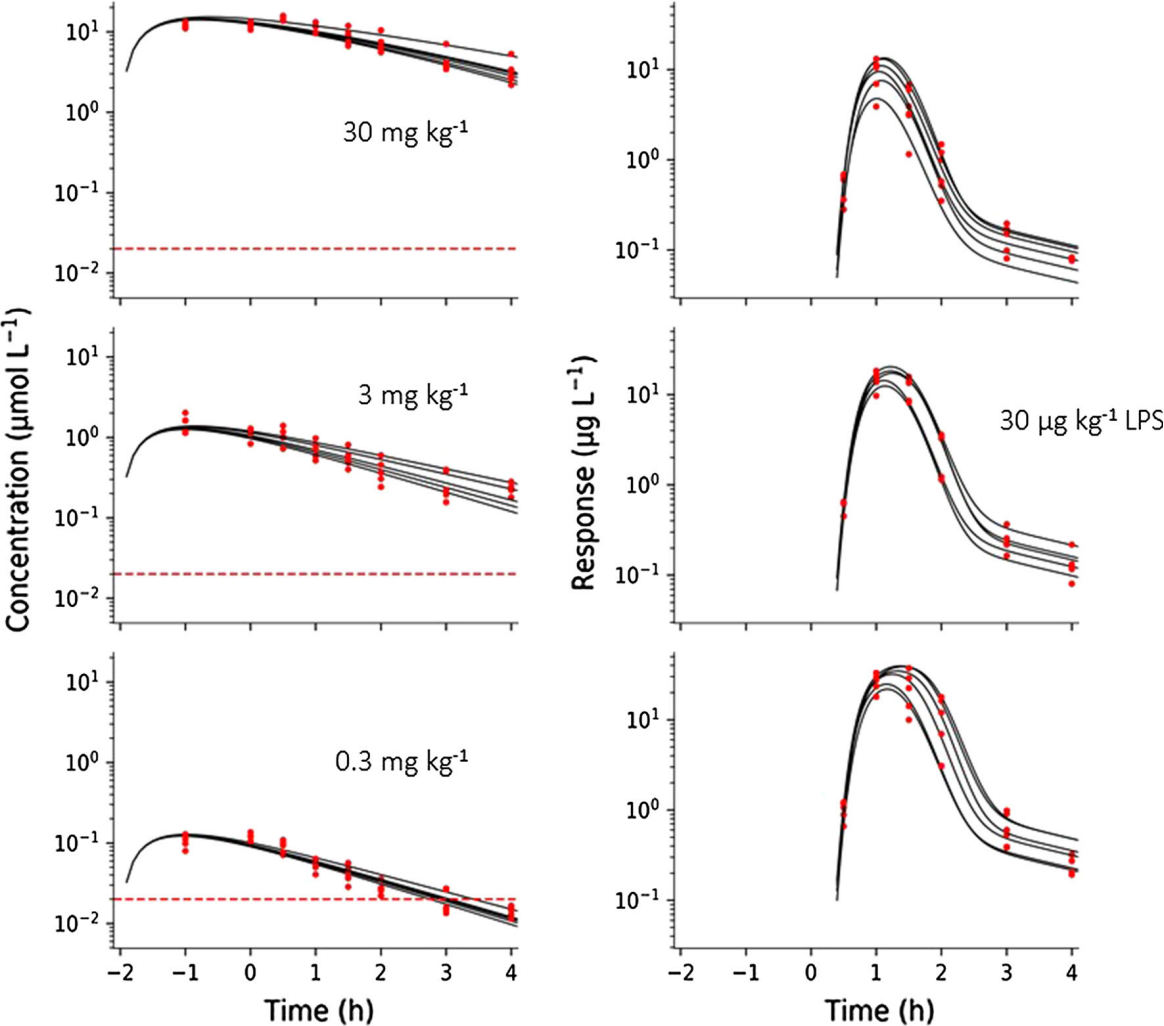
Left column: Observed (red dots) and model-predicted (solid lines) concentration–time data of test compound (A) of all subjects in Study 2. Right column: Observed (red dots) and model-predicted (solid lines) TNF_α_-response data of all subjects in Study 2. TNF_α_-response was observed after a fixed LPS challenge of 30 µg·kg^−1^. Test compound doses were 0.3 mg·kg^−1^ (upper row), 3 mg·kg^−1^ (middle row), and 30 mg·kg^−1^ (bottom row) (Color figure online)

The exposure to test-compound A was well characterized by Eqs. 1, 2 (Fig. 9 left). Test compound was given 2 h prior to the LPS challenge dose (30 µg·kg^−1^). The model-predicted test compound concentration peaked within an hour at the lowest dose (0.3 mg·kg^−1^), consistent with experimental data. A peak shift was then observed in model predictions due to the capacity-limited elimination with increasing doses of test compound (at 3, 30 mg·kg^−1^). All pharmacokinetic parameters and their precision were well characterized (Table 4).

**Table 4:** Final pharmacokinetic estimates, their CV% and IIV and CV%

Parameter	Units	Final estimate	CV%	IIV% (CV %)	IIV CV%
*k*_*a*_	h^−1^	1.72	12	–	–
*V*_*p*_	L·kg^−1^	3.30	4.2	–	–
*V*_max_	µmol·h^−1^·kg^−1^	32.2	14	11.5	22
*K*_*m*_	µmol·L^−1^	18.2	16	–	–

The model captured all features (such as onset, intensity and duration) of the TNF_α_-response at a fixed LPS challenge (30 µg·kg^−1^) and varying test compound doses (Fig. 9 right). A slight leftward shift in TNF_α_ peak response was observed for increasing test compound doses. The final test compound parameters of *I*_*max*_ and *IC*_*50*_ are shown in Table 5. Test compound displayed partial inhibition (*I*_*max*_ = 0.675 or 68%) of LPS-induced TNF_α_-response, and a corresponding potency of about 20 nmol·L^−1^ (*IC*_*50*_ = 23.1 nM) as total plasma concentration of test compound A.

**Table 5:** Final pharmacodynamic model estimates, their CV% and IIV and IIV CV%

Parameter	Units	Final estimate	CV%	IIV%	IIV CV%
*I*_*max*_	–	0.675	5	25.1	68
*IC*_*50*_	nmol·L^−1^	23.1	26	24.5	127

#### Between-subject variability and residual uncertainty

**Fig. 10:**
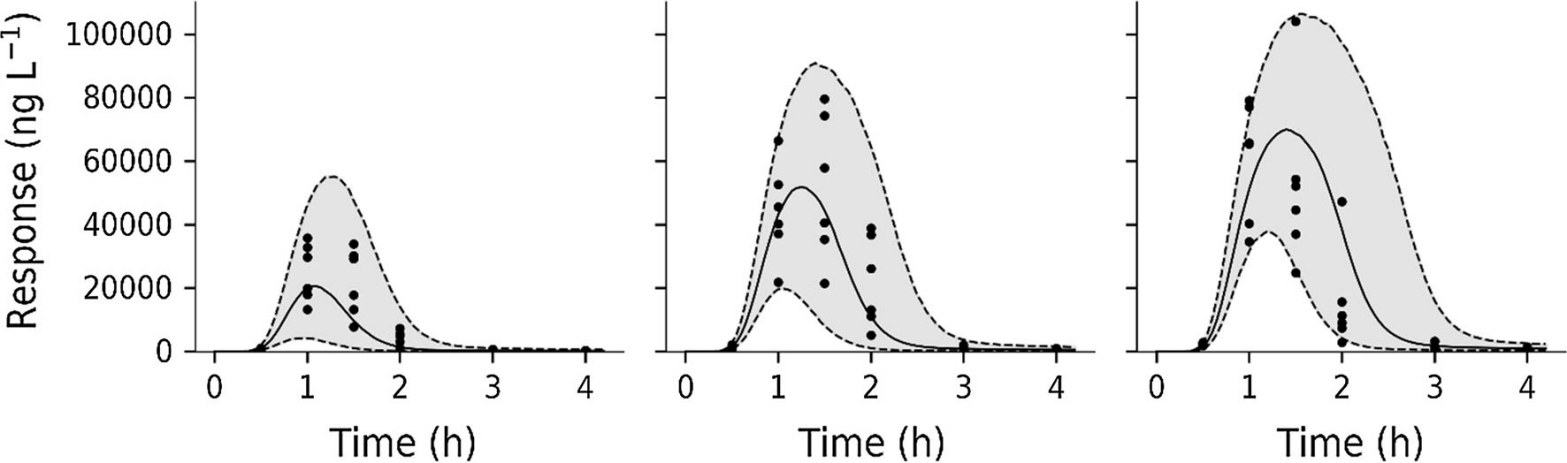
Visual predictive checks for the TNF_α_-response during LPS challenge (Study 1). Solid black symbols correspond to model-predicted time courses. The shaded grey areas show variability in predicted time courses. Dashed lines show the 5% (lower) and 95% (upper) percentiles and the middle solid line is the median. LPS challenge was 3 µg·kg^−1^ (left), 30 µg·kg^−1^ (middle) and 300 µg·kg^−1^ (right)

**Fig. 11:**
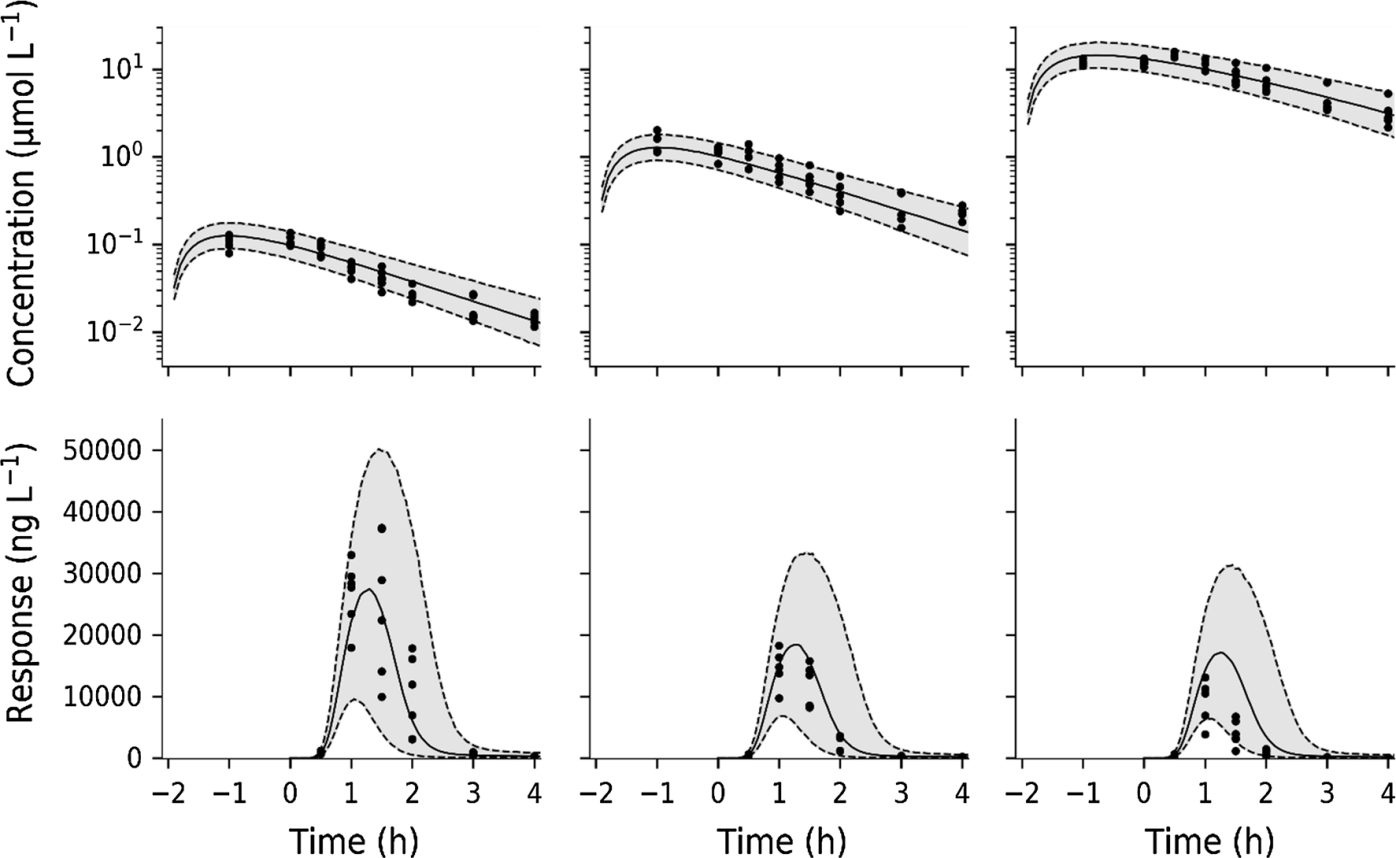
Semi-logarithmic plot of test compound exposure and TNF_α_-response (Study 2). Upper row: Visual predictive check of test compound concentration. Lower row: Visual predictive check of TNF_α_-response at 30 µg·kg^−1^ LPS challenge and varying test compound intervention. Filled circles correspond to model-predicted time courses for test-compound concentration and TNF_α_-response, respectively. The shaded grey areas are the predicted variability. Dashed lines show the 5 to 95% percentiles and solid line the median. Test compound doses were 0.3 mg·kg^−1^ (left column), 3 mg·kg^−1^ (middle column) and 30 mg·kg^−1^ (right column)

The inter-individual variability in TNF_α_-response (Study 1) is well predicted in the 3 and 30 µg·kg^−1^ dose groups (Fig. 10 left and middle). The inter-individual variability in TNF_α_-response is also well predicted in the 0.3 mg·kg^−1^ test compound dose group (Fig. 11 lower left). Variability is overestimated in the 3 and 30 mg·kg^−1^ dose group (Fig. 11 lower middle and right).

### Model simulations

**Fig. 12:**
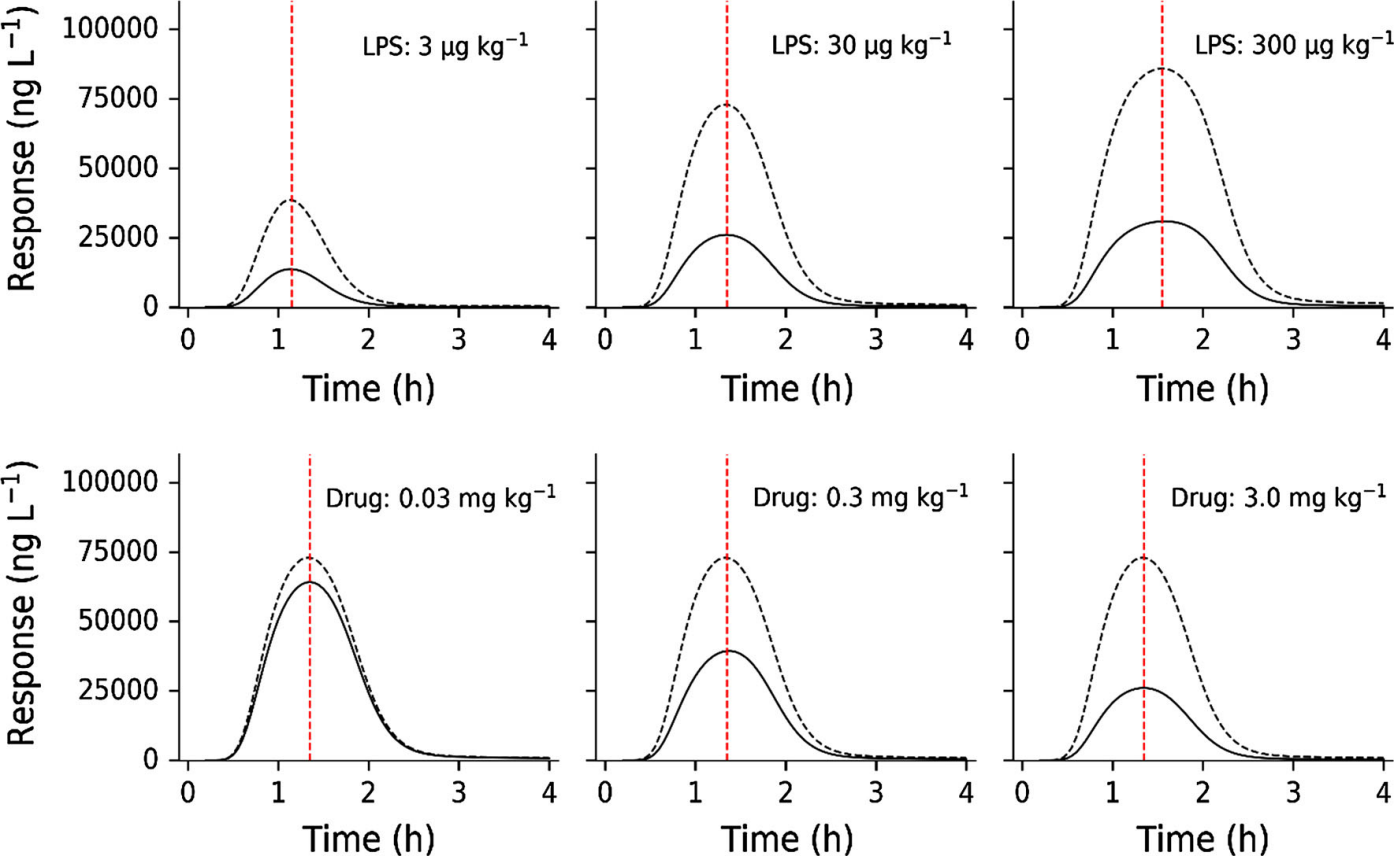
Upper row: Impact of different LPS doses (3, 30, 300 µg·kg^−1^) on TNF_α_-response given with (solid lines) and without (dashed line) 3 mg·kg^−1^ of test compound. Bottom row: Impact of a fixed LPS dose (30 μg·kg^−1^) on TNF_α_-response with (solid lines) and without (dashed lines) changing test compound doses (0.03, 0.3 and 3.0 mg·kg^−1^). The red vertical dashed lines show the peak-time locations (Color figure online)

Model simulations were done with a fixed test compound dose (3 mg·kg^−1^) and increasing LPS challenges (Fig. 12, upper row) in order to clarify the behavior of the model. Predictions show suppression of TNF_α_ peak response proportional to LPS challenge, as well as a peak-shift in TNF_α_-response with increasing LPS doses. Model simulations were also performed with a fixed challenge dose (30 µg·kg^−1^) and varying test compound doses (0.03, 0.3 and 3.0 mg·kg^−1^) (Fig. 12, bottom row). Approximately 70% suppression was observed in TNF_α_-response with the 3.0 mg·kg^−1^ dose since *I*_*max*_ was estimated to 0.675. The model-predicted in vivo potency *IC*_*50*_ of test compound is 20 nM (Table 3), which is consistent with experimental data. The test compound exposure covers a 10 to 1000 nM concentration range, which brackets the potency estimate.

## Discussion

A mechanism-based model describing TNF_α_-response was fitted to data obtained after several LPS challenges alone (Study 1) and a fixed LPS challenge in combination with varying doses of test compound (Study 2). The model captured experimental data well and gave accurate and precise parameters. *“What*-*if”* predictions were then made to explore model behavior at a fixed test compound dose and varying LPS challenges, and the reverse scenario. This was done to further evaluate the combined impact of test-compound and LPS challenge on the time course of TNF_α_ with respect to lag-times, peak-shifts and duration response.

### Experimental data

Test compound is a phosphodiesterase 4 PDE4 inhibitor, which indirectly targets mechanisms responsible for TNF_α_ release. This requires the compound to be present during LPS challenge, since it does not affect circulating TNF_α_, which has been shown previously. Test compound demonstrates partial inhibition of TNF_α_ release. Experimental data of test compound exposure were lacking prior to *C*_*max*_ with a predicted *t*_*max*_ at about 1 to 2 h (Fig. 9).

The biological mechanism behind LPS challenge on TNF_α_ release was described previously [23], and a model was therefore constructed to capture that behavior (Fig. 2). Saturable stimulation of TNF_α_ release was seen for the 0.3–30 µg·kg^−1^ LPS dose range (Fig. 6 left), which is also supported by other studies [9]. There seems to be a fixed time-delay in the onset of TNF_α_-response of approximately 30 min after the LPS challenge (Fig. 5), which suggests a saturable stimulatory effect of LPS. Similar studies [6, 10–12] have also captured the time-delay before onset of TNF_α_-response in plasma. Low variability was seen in exposure to test compound, which was captured by the model (Fig. 8 left and Fig. 10 upper row).

Following onset of TNF_α_-response, the rise of TNF_α_ occurred rapidly (Fig. 5 left) and displayed a peak-shift with increasing LPS challenge (Fig. 11 upper row). The rapid rise and decline of TNF_α_ indicates a high turnover. The extent of drug exposure will then govern the duration of TNF_α_-response. The current mechanism of action will not allow sufficient inhibition of TNF_α_ release in acute treatment. Both transcriptional and post-transcriptional mechanisms related to test compound [8, 9] have been suggested to influence the TNF_α_ release.

### Model regression

Acute cytokine release has previously been modeled with discontinuous functions, allowing the induced formation of TNF_α_ to take place only for a defined period [10–12, 24]. Others have used a continuous model as stimulatory function coupled to transit compartments [6]. However, the present design revealed that following a dose-independent time-delay of 30 min, rapid onset of response and saturable intensity was observed in the TNF_α_-response. The intensity of response was then followed by a bi-phasic terminal decline in TNF_α_-response. The bi-phasic decline was captured by a 2-compartment TNF_α_-response model (Fig. 2). A multi-phasic decline of TNF_α_-response coupled to rebound after LPS provocations has also been reported for primates [6]. In contrast to the primate study, no rebound was seen in TNF_α_-response in Sprague–Dawley rats.

The estimated fractional turnover rate of TNF_α_ is 5.65 h^−1^ in Sprague–Dawley rats, which is consistent with 0.5–4.51 h^−1^ in mice, 10 h^−1^ in cynomolgus monkeys and 1.82 h^−1^ in humans [6, 10–12, 24, 28]. This suggests a fast turnover or short half-life of TNF_α_ in all studied species so far. The transfer rate *k*_*s*_ was estimated to about 3.3 h^−1^, which leads to a delay of peak stimulation of about 1.2 h (Figs. 8 and 9 right). Previously published data support this, reporting TNF_α_ peak concentrations between 1 and 2 h after LPS administration, independently of species [6, 10–12, 24].

The Michaelis–Menten constant of test compound clearance was predicted to be 20 µM (18.2 μmol·L^−1^, Table 4), which is 1000-fold higher than its potency *IC*_*50*_ (Table 5). Test compound exposure in the 30 mg·kg^−1^ highest group barely reached 20 µM but stayed close to 10 µM for about 2 h before starting the decline at a slower rate than in the low dose (0.3 mg·kg^−1^ A) group. The Michaelis–Menten equation suggests that saturation of elimination is occurring and mechanistically this might be due to saturation of drug-metabolizing enzymes and/or drug transporter.

Physiologically, there is no observable baseline concentration of TNF_α_ in blood. The cytokine is only released into blood from activated monocytes in response to an immunological stimulus [29]. The drug-induced inhibition acts on the LPS stimulatory function *S(LPS)*.

The model estimated test compound potency *IC*_*50*_ is about 20 nM (0.0231 μmol·L^−1^, Table 5) which is consistent with the exploratory data on TNF_α_-response (Fig. 7). The importance of also incorporating a vehicle control group improves the assessment of how inter-occasion variability may impact, for example, potency and efficacy assessment in highly variable data. Ideally, all substudies should contain vehicle control group(s). The final estimate of *I*_*max*_ suggests that there is a partial reduction in TNF_α_-response of 70% at the highest test-compound dose of 30 mg·kg^−1^.

We would also like to highlight the importance of actually measuring the challenger as such, rather than making indirect inferences about its behavior via a biophase model [6]. LPS exposure data would be helpful in future studies to examine whether the inter-individual variability observed in TNF_α_-response is explained by a variable LPS exposure or not. The uncertainty in the actual LPS exposure will indirectly inflate how accurate and precise the drug parameters, such as *IC*_*50*_ and *I*_*max*_, are estimated.

Vehicle control data (combined with test compound dose 0 mg·kg^−1^) of TNF_α_-response were lacking in Study 2. Therefore, fixed final parameter values of system properties (Study 1) were used to facilitate the regression of test compound specific *I*_*max*_ and *IC*_*50*_ parameters of Study 2. A crossover design measuring TNF_α_-response following the same LPS challenge with or without drug intervention in each subject may be considered in future designs.

### Model simulations

Model simulations were done with a fixed test compound dose (3 mg·kg^−1^) and increasing LPS challenges (Fig. 11, upper row) in order to illuminate the determinants of onset, intensity and duration of TNF_α_-response. Predictions show suppression of TNF_α_ peak response proportional to LPS challenge, as well as a peak-shift in TNF_α_-response with increasing LPS doses. Model simulations were also performed with a fixed challenge dose (30 µg·kg^−1^) and varying test compound doses (0.03, 0.3 and 3.0 mg·kg^−1^) (Fig. 11, bottom row). Approximately 70% suppression was seen in TNF_α_-response with the 3.0 mg·kg^−1^ dose because *I*_*max*_ was estimated to 0.675. Model-predicted in vivo potency *IC*_*50*_ of test compound is 20 nM (Table 3), which is consistent with experimental data. The test compound exposure covers the 1.0 to 100 nM concentration range, which brackets the potency estimate. Multiple LPS challenges demonstrated a peak-shift in TNF_α_-response with increasing doses.

LPS exposure should, if possible, be incorporated into future studies to handle the origin of variability seen in TNF_α_ response. Information about the onset, intensity and duration of TNF_α_ response upon LPS challenge and/or test compound intervention may be improved by higher resolution of experimental TNF_α_ response data at pivotal time points [6]. Repeated (sparse) sampling of TNF_α_ response in the same individual after LPS or test compound intervention is still recommended. Table 6 contains a summary of major findings related to the pharmacodynamic time course and suggested improvements of future designs of TNF_α_ response as a biomarker. Table 7 is an attempt to summarize some general points to consider related to topics such as potency, experimental design and target biology.

**Table 6:** Summary of major findings and suggested improvements for future designs

Study	Points to consider	Major findings	Suggested improvement of design
1	Delay after LPS dose	Constant delay of 30 min independently of LPS dose	Sampling of systemic LPS exposure. Design(s) for understanding your target. Apply a biophase model of LPS exposure
1	Peak-shifts in TNF_α_-response	Peak-shifts in TNF_α_-response seen with increasing doses of LPS	Sampling of systemic LPS exposure, which may explain some of the peak-shift in TNF_α_-response
1	Assessment of saturation of response	Saturation of TNF_α_-response at higher LPS doses assessed from dose-normalized TNF_α_-response time courses	Sampling of systemic LPS exposure, which may explain some of the saturation observed in TNF_α_-response
2	Baseline response	Vehicle control group was lacking in Study 2 to assess the impact of LPS challenge on TNF_α_-response	Sampling of systemic LPS exposure. Use cross-over design for determination of LPS-induced TNF_α_-response with and without test compound
2	Delay after test compound dose	Delay in onset of TNF_α_-response upon drug intervention is not observed	Allow higher granularity of test compound exposure
2	Peak-shifts	No peak-shifts were seen in LPS induced TNF_α_-response with increasing test compound doses	Sampling of systemic LPS exposure, which may explain lack of peak-shift in TNF_α_-response with increasing test compound doses
2	Saturation of response	A maximum inhibitory effect of test compound was obtained in the TNF_α_-response at an LPS challenge dose of 30 µg·kg^−1^	Sampling of systemic LPS exposure, which may explain some of the saturation observed in TNF_α_-response with increasing test compound doses

**Table 7:** Points to consider when modelling cytokine challenge test data

Topic	Points to consider
General	High interest in modelling challenge test data, but a robust quantitative approach is still in its infancy. More diverse datasets and models are needed
Potency	Drug screening and clinical efficacy are primarily driven by in vitro and ex vivo whole-blood (WB) assays where cytokine release is measured after LPS challenge in vitro. The primary questions relate to the predictive power of WB assays. What is the role of blood-born *versus* tissue-born cells? The in vivo*/*in vitro correlation IVIVC may give some guidance with respect to potential clinical outcome, where only WB is available at an early stage. The IVIVC with respect to biochemical target may also exclude off-target effects *IC*_*50*_ is approximately 20 nM. In vivo potency is a conglomerate of binding (affinity, *k*_*off*_ and *k*_*on*_), target turnover (*k*_*deg*_) and ligand-target complex kinetics (*k*_*e(RL)*_) [32, 33]. This new expression enables a more efficient species-to-species comparison of pharmacodynamic properties. *I*_*max*_ gives insight about whether full or partial TNF_α_ suppression is possible (tissues responding to LPS but lacking the target)
Study design	The TNF_α_-response is rapid and transient, which is a challenge in experimental design. Small time-differences may result in large baseline observations, and therefore cause erroneous assessment of drug inhibition. This is an argument against single (end) point studies and favor biomarker time courses. Vehicle control groups should be included in all substudies
Target biology	Can a mechanism-based model cast light on LPS acting on precursor pool-driven release of TNF_α_ (post-translational effects) or mRNA-driven induction (transcriptional effects)? The onset of TNF_α_ release is rapid, suggesting post-translational mechanisms. Can better insight be accomplished (depletion of precursor pool/efficacy after repeated dosing)? Is there a risk of tachyphylaxia with either mechanism?
Dosing regimens	Is the drug mechanism curative or prophylactic? If prophylactic, how should the dose be given optimally, and what are the pharmacokinetic requirements? What is the translational potential of the model across species? Are human systems parameters predictable from animal data?

## Overall conclusion

A mechanism-based biomarker model of TNF_α_-response, including different external provocations of LPS challenge and test compound intervention, was developed to serve as a modelling tool. The model contained system properties (such as *k*_*t*_*, k*_*out*_), challenge characteristics (such as *k*_*s*_*, k*_*LPS*_*, K*_*m, LPS*_*, S*_*max*_*, SC*_*50*_) and test- compound-related parameters (*I*_*max*_*, IC*_*50*_). The exposure to test compound was modelled by means of first-order input and Michaelis–Menten type of nonlinear elimination. Test compound potency was estimated to 20 nM with a 70% partial reduction in TNF_α_-response at the highest dose of 30 mg·kg^−1^. Future selection of drug candidates may focus the estimation on potency and efficacy applying the selected structure consisting of TNF_α_ system and LPS challenge characteristics. A related aim was to demonstrate how an exploratory (graphical) analysis may guide us to a tentative model structure, which enables us to better understand target biology. The analysis demonstrated how to tackle a biomarker with a baseline below the limit of detection. Repeated LPS-challenges may also reveal how the rate and extent of replenishment of TNF_α_ pools occur. Lack of LPS exposure-time courses was solved by including a biophase model, with the underlying assumption that TNF_α_ response time courses as such contain kinetic information. A transduction type of model with non-linear stimulation of TNF_α_ release was finally selected. Typical features of a challenge experiment were shown by means of model simulations. Experimental shortcomings of present and published designs were identified and discussed. The final model coupled to suggested guidance rules may serve as a general basis for the collection and analysis of pharmacological challenge data of future studies.

### Electronic supplementary material

Below is the link to the electronic supplementary material.

Supplementary material 1 (DOCX 340 kb)

## Figures and Tables

**Fig. 13:**
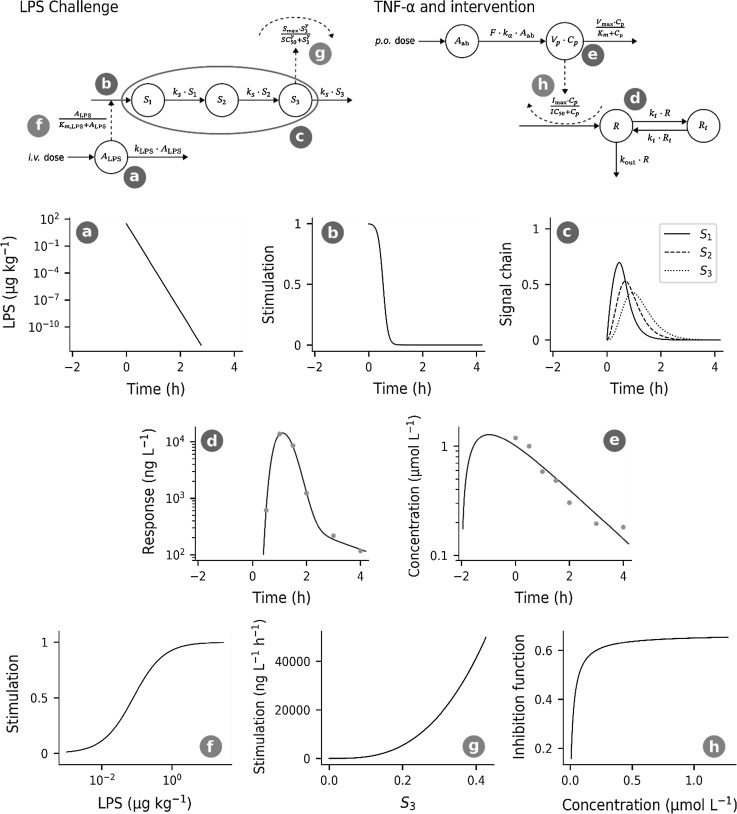
Presentation of time courses and auxiliary relationships in the model for a single subject after LPS challenge (30 μg·kg^−1^) and test-compound administration (3 mg·kg^−1^). Blue labels mark concentration–time courses, whereas red labels mark relationships between different quantities in the model. LPS biophase concentration is shown in plot *a*, the time-dependent stimulation of signal compartment *S*_*1*_ in plot *b* and the concentrations in the three signal compartments *S*_*1*_ to *S*_*3*_ in plot *c*. In plots *d* and *e*, showing response and test compound concentration, observed data is included. Plot *f* shows the relationship of concentration in the LPS biophase and the stimulation of signal compartment *S*_*1*_. In plots *g* and *h* the stimulation and inhibition of the TNF_α_-response are shown over concentration in compartment *S*_*3*_ and drug concentration *C*_*p*_, respectively (Color figure online)

## Appendix 1

### Principal parts of the model

The model described in Eqs. 1–7 was formulated based on five central observations in data, namely (1) LPS dose-independent delay of onset of TNF_α_-response; (2) LPS dose-dependent duration of TNF_α_-response; (3) LPS challenge has a saturable stimulatory impact on the TNF_α_-response; (4) test compound has a saturable inhibitory impact on the TNF_α_-response; (5) TNF_α_-response declines in a bi-phasic fashion post-peak.

The onset of TNF_α_-response was delayed about 30 min independently of LPS challenge dose (Fig. 5, left). The combination of a time-limited constant input signal, transportation through a chain of delay compartments and a nonlinear (sigmoid) stimulatory function captures data nicely for all LPS challenges. Using a first-order input/output biophase model for the LPS kinetics (Eq. 3; Fig. 13a) combined with a saturable stimulatory function (Eq. 4; Fig. 13b) gave constant time-limited stimulatory input of TNF_α_. Higher LPS doses will increase the time during which the input signal is totally saturated, which explains the LPS dose-dependent duration of TNF_α_-response. This input signal is transported through a series of transduction compartments (Eq. 4; Fig. 13c), which explains the delay of onset of TNF_α_-response. Stimulation of TNF_α_ release is then captured by means of a saturable function (Eq. 5; Fig. 13g), which explains the rapid onset of TNF_α_-response.

The saturable stimulatory impact of LPS challenge on TNF_α_-response is summarized in Fig. 6 (left), which is described by Eq. 5. Increasing the dose of test-compound had a nonlinear inhibitory effect on TNF_α_-response (Fig. 6, right), which is captured by Eq. 6. The post-peak bi-phasic decline in TNF_α_-response (Fig. 5, right) was modelled by inclusion of a peripheral response compartment *R*_*t*_ (Eq. 7).

### Computational details

#### Parameter selection for NLME modelling

To determine which parameters had a large influence on the model-predicted TNF_α_ concentration, a variance-based sensitivity analysis was conducted using the Sobol method [14]. The Python (Python Software Foundation, https://www.python.org, version 3.6) package SALib [15] was used for this analysis. Additionally, complementary roles of parameters (e.g. *S*_*max*_ and *SC*_*50*_ both influence peak TNF_α_ concentration) were considered and parameters exhibiting larger IIV during test runs were preferred when deciding what parameters to associate with IIV or not for NLME modelling. Both *I*_*max*_ and *IC*_*50*_ were of great interest in this study and were therefore modelled with IIV. Resulting from this analysis, the following distributions were assigned to parameters: *V*_max_ (Eq. 2) normally distributed, *k*_*LPS*_ (Eq. 3), *SC*_*50*_ (Eq. 5) and *k*_*out*_ (Eq. 7) and *IC*_*50*_ (Eq. 6) log-normally distributed, and *I*_*max*_ (Eq. 6) logit-normally distributed. No correlations between random effects were included in the model.

#### Identifiability analysis

A basic prerequisite for parameter estimation of a complex model is for the model to be structurally identifiable, given observed variables. Structurally identifiable means that any two distinct sets of parameters of a model will not result in identical observations, i.e., parameter values are uniquely determined by observed data. The model Eqs. 1–7 were checked for local structural identifiability using the Exact Arithmetic Rank (EAR) algorithm [16–18]. The Wolfram Mathematica (Wolfram Research Inc., Version 11.1) package *IdentifiabilityAnalysis* (see Karlsson et al. [17] for a theoretical description; implemented at the Fraunhofer-Chalmers Centre) was used for this analysis. The basic EAR algorithm requires the model equations to be rational functions. However, a larger class of system can be addressed by EAR by transformations demonstrated in Reference [19].

#### Model and convergence checking

Parameter convergence was assessed by repeated estimation using different initial values for parameters leading to sets of parameter estimates in close proximity to each other. Standard errors, derived from a stochastic approximation of the Fisher information matrix, were as low as possible. Individual parameters were simulated from the conditional parameter distribution and statistical parameter models were checked by comparison of simulated individual parameters to the theoretical distributions [21]. Model fit was assessed through the investigation of individual and population residuals and their distributions as well as visual predictive checks [22].

## Appendix 2

### Contributions from inflammation and drug intervention at equilibrium

Model simulations using Eq. 8 showing the joint impact of exposure (*C*_*p*_) and LPS stimuli (*S*_*3*_) on TNF_α_-response (left), and exposure (*C*_*p*_) and biophase amount of LPS (*A*_*LPS*_) on TNF_α_-response (right).
