# Supplementary material for: Challenge model of TNFα turnover at varying LPS and drug provocations
Source: J Pharmacokinet Pharmacodyn. 2019 Feb 18;46(3):223–40. doi: 10.1007/s10928-019-09622-x (PMC6529397; doi:10.1007/s10928-019-09622-x)
Supplement: Supplementary file 1 — Supplementary material 1 (DOCX 340 kb) [file 10928_2019_9622_MOESM1_ESM.docx]

Supplementary for “Challenge model of TNF_α_ turnover at varying LPS and drug provocations”

## Authors

Felix Held^1,2,5^, Edmund Hoppe^3^, Marija Cvijovic^2^, Mats Jirstrand^1^, and Johan Gabrielsson^4^

## Affiliations

1. Fraunhofer-Chalmers Centre, Chalmers Science Park, Gothenburg, Sweden
2. Department of Mathematical Sciences, Chalmers University of Technology and University of Gothenburg, Gothenburg, Sweden
3. Grünenthal GmbH, Aachen, Germany
4. Swedish University of Agricultural Sciences, Department of Biomedical Sciences and Veterinary Public Health, Box 7028, 75007 Uppsala, Sweden, [johan.gabrielsson@slu.se](mailto:johan.gabrielsson@slu.se)
5. Address correspondence to [felix.held@chalmers.se](mailto:felix.held@chalmers.se)

# Results for variance-based sensitivity analysis

It was necessary to decide which parameters in the PK/PD model (Equations 1‑5) should be modelled with IIV. To support this decision process a variance-based sensitivity analysis was conducted using the Sobol method [1]. The Python (Python Software Foundation, https://www.python.org, version 3.6) package SALib [2] was used for this analysis. Additionally, complementary roles of parameters (e.g. *S*_max_ and *SC*_50_ both influence peak TNF_α_ concentration) were considered and parameters exhibiting larger IIV during test runs were preferred when deciding what parameters to associate with IIV or not for NLME modelling.

*Total sensitivity S_T_* is a measure for the total influence of the respective parameter on the variability of the model output. This can include interactions of the respective parameters with other parameters that have an influence on the model output. *First-order sensitivity S_1_* measures how much of total sensitivity S_T_ can be explained by varying just the respective parameter without interactions with other parameters.

The sensitivity analysis for the PK model (Equations 1-2) in Fig. S1 revealed that *V*_max_ had most influence on the variability of drug plasma concentration *C_p_.* No other parameter seemed to have similar influence and *V*_max_ was chosen as the only parameter with IIV in the PK model.

The sensitivity analysis for the PD model (Equations 3-5) was less unambiguous. In Fig. S2 it can be seen that *k*_LPS_, *k_s_*, *S*_max_, *SC_50_*, and *k*_out_ have the largest influence on the variability of TNF_α_ response. However, initial parameter estimation runs revealed little IIV in *k_s_* and *S*_max_. These were therefore dropped to improve numerical accuracy for the other parameters.


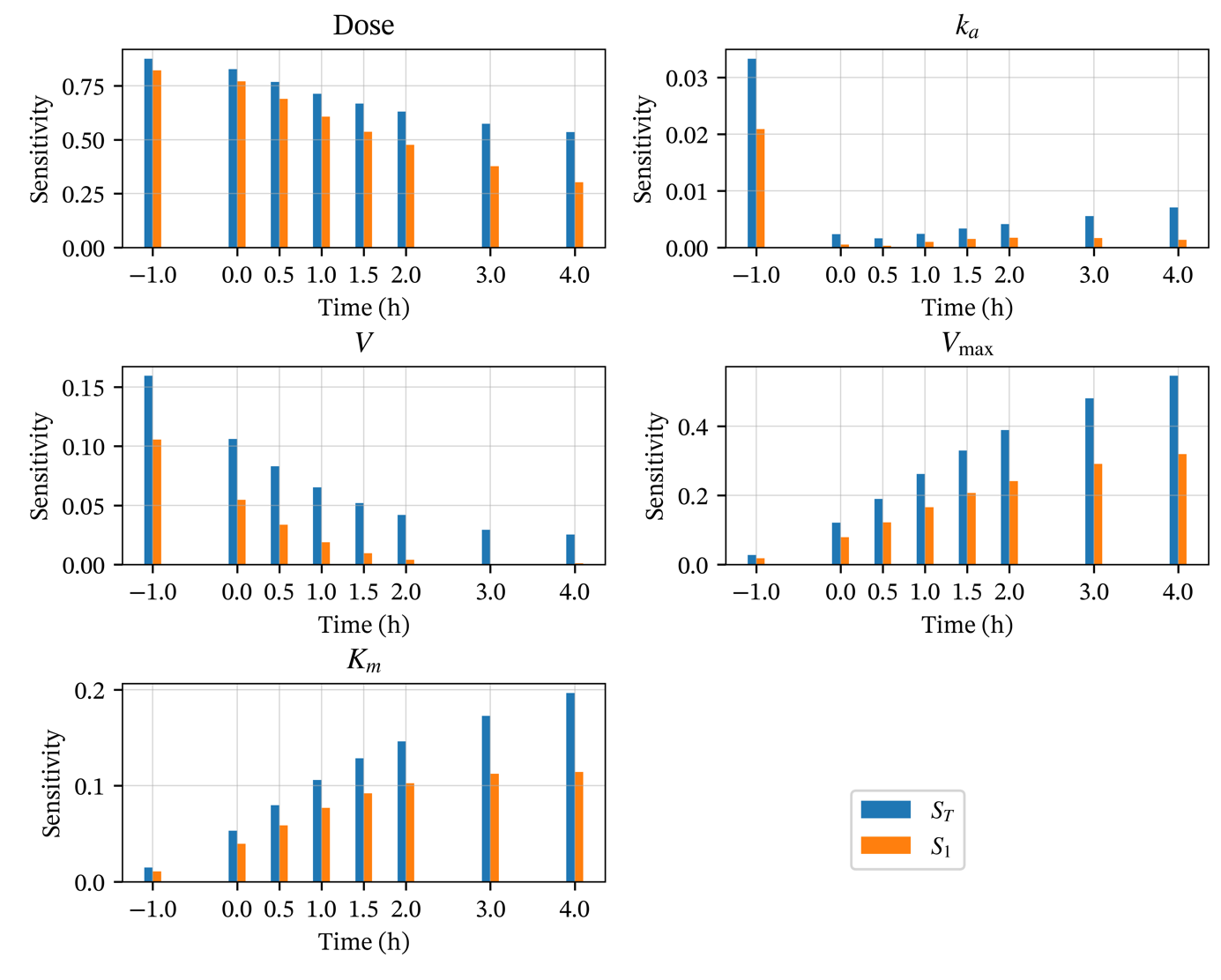


**Fig. S1** Total sensitivities S_T_ and first-order sensitivities S_1_ for drug concentration in the plasma compartment (Equations 1-2) at the time points used during data collation. Dose refers here to administrated dose of Test compound A


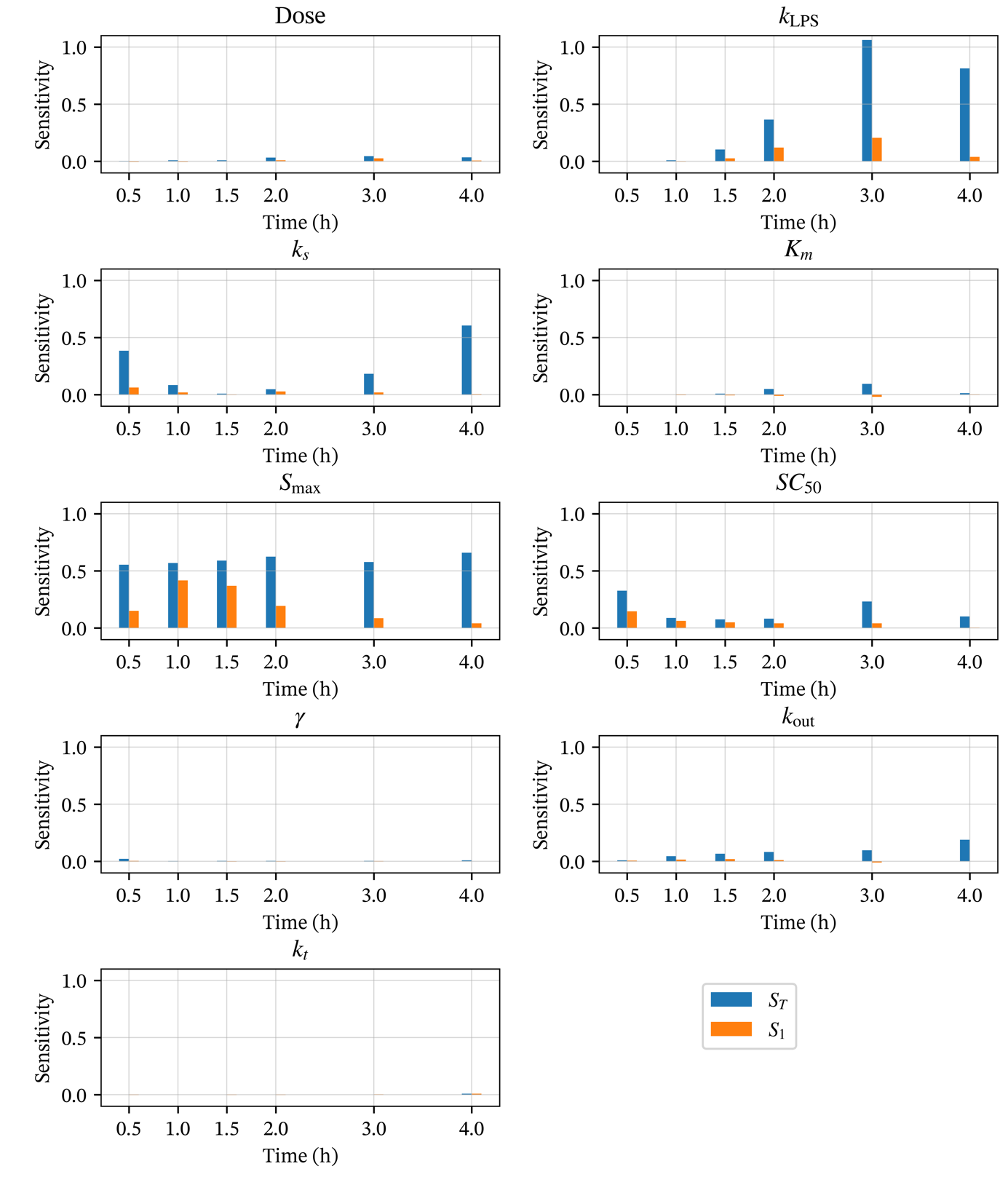


**Fig. S2** Total sensitivities S_T_ and first-order sensitivities S_1_ for the TNF_α_-response after LPS-stimulation (Equations 3‑5) at the time-points used during data collection. Dose refers here to administrated LPS amount

# References

1. Saltelli A, Ratto M, Andres T, Campolongo F, Cariboni J, Gatelli D, Saisana M, Tarantola S (2008) Global sensitivity analysis. The Primer. John Wiley & Sons, Chichester, England

2. Herman J, Usher W (2017) SALib: An open-source Python library for sensitivity analysis. J Open Source Softw 2. <https://doi.org/10.21105/joss.00097>
